# Knowledge, Attitudes, Practices and Education Needs of Dentists on Domestic Violence: A Systematic Review

**DOI:** 10.1111/jep.70438

**Published:** 2026-04-23

**Authors:** Sthefanie del Carmen Perez Puello, Vitor Rafael Gomes, Brunna Verna Castro Gondinho, Júlia Vitório Octaviani, Eva Zafra Aparici, Luciane Miranda Guerra

**Affiliations:** ^1^ Departamento de Ciências da Saúde e Odontologia Infantil, Faculdade de Odontologia de Piracicaba (FOP/UNICAMP), Piracicaba‐SP Universidade Estadual de Campinas, Faculdade de Odontologia de Piracicaba São Paulo Brasil; ^2^ Universidade Estadual do Piauí, Faculdade de Odontologia e Enfermagem (FACOE—UESPI) Departamento de Saúde Coletiva Piaui Brasil; ^3^ Universidad Rovira i Virgili (URV) Department of Anthropology, Philosophy and Social Service Tarragona Spain

**Keywords:** health care, systematic review, violence against women

## Abstract

The objective of the present systematic review was to evaluate the scientific evidence on the knowledge, attitudes, practices level (KAP) and education needs among dentists in the identification of clinical signs and notification of domestic violence (DV). Electronic searches were performed in PubMed/Medline, ScienceDirect, EBSCOHost, Scopus databases, and grey literature using the keywords ‘Domestic Violence’, ‘Abused Women’, ‘Dentistry’, ‘Oral health’, ‘Women's Health Services’ and ‘Health Knowledge, Attitudes, Practice’. Cross‐sectional or longitudinal studies, without restriction on publication year, published in English, using calibrated or validated questionnaires to assess the dentist KAP level and education needs for identification of clinical signs and notification of DV were considered. The Ottawa Scale was applied to assess the risk of bias. In the final analysis, 5 studies were included, conducted in the United States, France, Brazil and Pakistan. The total sample was 1735 general and specialist dentists. All studies showed that the participants had a low level of CAP in identifying clinical signs and reporting DV. In addition, 27% reported having identified at least one case in clinical practice. Most of participants stated that they had not received training on care for victims of DV. The studies had a low risk of bias. Scientific evidence suggests that dentists have a low level of KAP in the identification of clinical signs and notification of DV and have a high need for education in this area.

## 1 Introduction

Globally, violence against women has been considered a public health problem due to the increased incidence of cases committed mainly by intimate partners. In addition, the COVID‐19 pandemic, together with social, economic, and psychological problems, has favoured the exposure of women and children to cases of domestic violence, producing an increase in the severity of injuries [1]. In Latin America, the armed groups, gangs, conflicts, and post‐conflict displacements have increased the violation of women's human rights and, consequently, favoured different types of violence, such as sexual, physical, economic, and patrimonial violence [2, 3].

According to the World Health Organisation (WHO), at least 35% of women in the world have suffered some type of violence (sexual or physical) by their partner [4]. Furthermore, more than 30% of femicides are perpetrated by the victim's partner [4]. In this sense, some studies report that a low educational level, belonging to family or social environments where violence is exercised as a form of control and patriarchal systems, as well as being in conditions of subordination, consuming alcoholic or hallucinogenic substances, are factors that, added to gender inequality, represent a greater risk for domestic violence [5, 6, 7].

In addition to the psychological and emotional consequences, at least 42% of victims of physical or sexual violence may suffer body injuries, mainly in the head, neck and extremities, such as lacerations, cuts, bruises, burns and in severe cases, fractures and even spontaneous abortions [8].

Considering the global deficiency in reporting cases of domestic violence, and that the head and neck region is the responsibility of dentists, they could play an important role in identifying, reporting and multidisciplinary approach to cases of domestic violence in the health system. However, when evaluating the tools used by dentists to assess dental and facial injuries caused by acts of domestic violence, it is concluded that there are protocols, but their effectiveness in identifying this type of injury has not been evaluated [9].

Femi‐Ajao et al. [10], in their study on the perception of women victims of domestic violence regarding the participation of the oral health team in their care, also reported that victims would accept being asked directly about injuries caused by the violence suffered. However, participants expressed doubts about the training of dentists regarding the protocols for approaching this area [10].

A study conducted in 8 Arab countries showed that more than 50% of dentists—both in the public and private sectors—are able to identify and intend to report cases that arise during consultations [11]. However, they report that their undergraduate or postgraduate training was insufficient to identify cases of violence and believe that efforts need to be redoubled to train dental professionals to approach victims in a humane way [12]. This was also demonstrated by Drigeard et al. [13] in a sample of French dentists, showing that more than 70% expressed interest in receiving information on the clinical characteristics and legal policies of victims of domestic violence [13]. Consequently, there is a need to train oral health professionals who besides high scientific knowledge and manual dexterity, are also aware of their active role in the emancipation and empowerment of women to prevent all types of violence, in addition to having knowledge about the identification and reporting protocols and approach to cases of domestic violence, acting decisively and avoiding the revictimization of women within the health system. Thus, the objective of this systematic review was to evaluate the current scientific evidence on the knowledge, attitudes and practices level and educational needs of dentists in identifying clinical signs and reporting cases of domestic violence.

## 2 Methodology

The items of the Preferred Reporting Items for Systematic Reviews and Meta‐Analysis (PRISMA) protocol were considered for the development of this systematic review. In addition, registration was carried out on the PROSPERO (Prospective Register of Systematic Reviews) platform.

### 2.1 Eligibility Criteria

The research question: what is the knowledge, attitudes, practices level and educational needs of dentists to identify clinical signs and reporting cases of domestic violence? This question was structured using the PECO strategy (Patients, Exposure, Comparison and Outcomes).

### 2.2 Inclusion Criteria

Cross‐sectional or longitudinal studies without restriction on publication year and published in English were considered. Studies that used calibrated or validated questionnaires to assess the level of knowledge, attitudes, practices and educational needs of dentists for identifying clinical signs and reporting domestic violence were also included.

### 2.3 Exclusion Criteria

Comprehensive literature reviews, clinical cases, book chapters, conference abstracts, and letters to the editor were excluded from the current systematic review.

### 2.4 Search Strategy

Searches were conducted in 2024 by two calibrated authors, considering the following databases: Medline/PubMed, ScienceDirect, Scopus, and EBSCO‐HOST. (Last update: September, 2025). The searches performed in the different databases are presented in Table 1. To identify additional articles that could meet the inclusion criteria of this systematic review, the reference list from selected articles and titles in the grey literature were read.

**Table 1:** Search strategy conducted in ScienceDirect, Scopus, Medline/Pubmed, and EBSCO‐HOST (September, 2025).

Medline/PubMed	
Strategy #1	Strategy #2
(((‘Domestic Violence’ OR ‘Abused Women’ OR ‘Women, Abused’)) AND ((‘dentistry’ OR ‘oral health’))) AND ((‘Women’ OR ‘Women's Health Services’)	(((‘Domestic Violence’ OR ‘Abused Women’ OR ‘Women, Abused’)) AND ((‘dentistry’ OR ‘oral health’))) AND ((‘Women’ OR ‘Women's Health Services’) AND (‘Health Knowledge, Attitudes, Practice’)
ScienceDirect	
Strategy #1	Strategy #2
Title, abstract or author‐specified keywords ((‘Domestic Violence’ OR ‘Abused Women’ OR ‘Women, Abused’) AND (‘dentistry’) AND (‘Women’ OR ‘Women's Health Services’))	Title, abstract or author‐specified keywords ((‘Domestic Violence’ OR ‘Abused Women’ OR ‘Women, Abused’) AND (‘dentistry’ OR ‘oral health’ OR ‘Dentists’) AND (‘Women’ OR ‘Women's Health Services’)AND (‘Health Knowledge, Attitudes, Practice’))
Scopus	
Strategy #1	Strategy #2
TITLE‐ABS‐KEY ((‘Domestic Violence’ OR ‘Abused Women’ OR ‘Women, Abused’) AND (‘dentistry’) AND (‘Women’ OR ‘Women's Health Services’))	TITLE‐ABS‐KEY (((‘Domestic Violence’ OR ‘Abused Women’ OR ‘Women, Abused’) AND (‘dentistry’ OR ‘oral health’ OR ‘Dentists’) AND (‘Women’ OR ‘Women's Health Services’) AND (‘Health Knowledge, Attitudes, Practice’)))
(‘Domestic Violence’ OR ‘Abused Women’ OR ‘Women, Abused’) AND (‘dentistry’) AND (‘Women’) AND (‘Women's Health Services’)	((‘Domestic Violence’ OR ‘Abused Women’ OR ‘Women, Abused’) AND (‘dentistry’ OR ‘oral health’ OR ‘Dentists’) AND (‘Women’ OR ‘Women's Health Services’) (‘Health Knowledge, Attitudes, Practice’))

### 2.5 Study Selection

Study selection was performed by two authors, who organised all article titles in spreadsheets according to the searches performed in each of the databases, using Microsoft Excel 2020 (Microsoft Corporation, Redmond, Washington, USA). Duplicate articles were eliminated and the remaining titles and abstracts were evaluated according to the eligibility criteria. Those that met the inclusion criteria were read in full text version. The evaluation of a third author was necessary in cases of disagreement on studies selection process.

### 2.6 Data Extraction

Data extraction was performed using tables to categorise the variables: author, publication year, country, type of study, sample (size, sex, age, educational level), sector in which participants work, questionnaire and method of application, variables evaluated, results on the knowledge, attitudes, practices level and educational needs among dentists in identifying clinical signs and reporting cases of domestic violence.

### 2.7 Risk of Bias

Considering that the included studies were cross‐sectional or longitudinal, the Newcastle‐Ottawa scale was used to assess the risk of bias of the selected studies [14]. The scale proposes the assessment of 3 sections, composed of 7 items to evaluate methodological aspects such as: sample selection (population characterisation, sample calculation) (3 points), comparability of population groups, adjustment of confounding factors (2 points) and outcome (assessment of the outcome, determination, formation and validity of the questionnaire and reported non‐response rate) (3 points). According to the score obtained, the selected studies were classified as low (4−8 points) or high (1−3 points) risk of bias.

### 2.8 Data Analysis

Data were extracted from the selected studies and assessed considering descriptive statistical parameters such as frequency and percentages. In addition, the associations reported in the studies were considered to answer the research question of the current systematic review. The different questionnaires and response categories implemented and the statistical tests used for their analysis highlighted the methodological heterogeneity of the studies, therefore, it was not possible to carry out a meta‐analysis.

## 3 Results

### 3.1 Search and Selection of Articles

**Figure 1:**
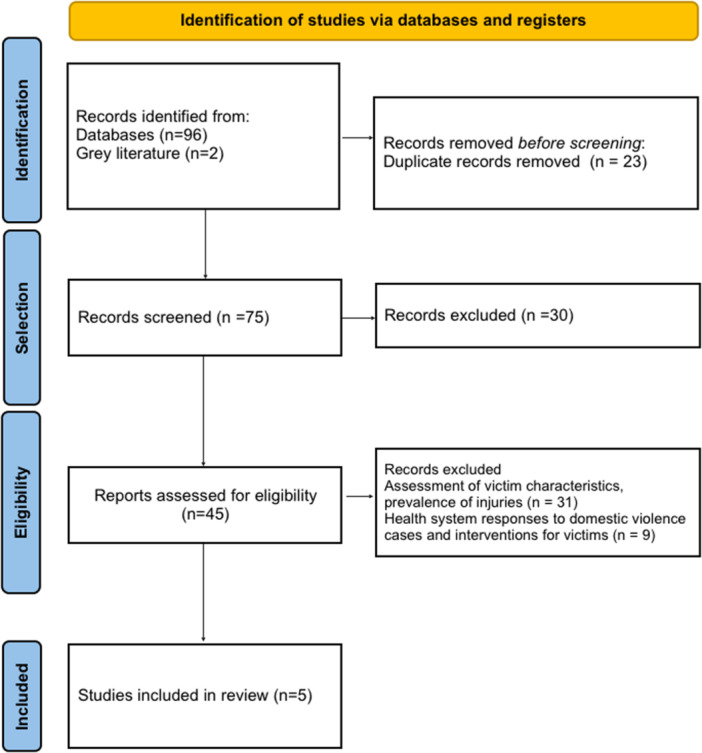
PRISMA flowchart for identification, selection, eligibility and included article (December, 2025). *Source*: Authors (2025).

Figure 1 shows the identification process, selection and inclusion of studies. In total, 98 articles were identified through electronic searches in databases and grey literature. Initially, 23 articles were duplicated and 30 articles were eliminated because they presented other thematic axes related to domestic violence. The titles and abstracts were read and after applying the eligibility criteria, 40 articles that addressed the assessment of the sociodemographic characteristics of victims, prevalence of injuries, protocols for response of health systems to cases and interventions for victims were excluded. In this sense, the qualitative synthesis included only 5 articles that met the inclusion criteria and reported the level of knowledge, attitudes, practices and educational needs among dentists about domestic violence. No time restriction was applied; however, the included studies were published between 2012 and 2021.

### 3.2 Characteristics of the Studies

The studies were published between 2012 and 2021, with populations from 12 countries, such as: United States (*n* = 117) [15], France (*n* = 418) [13], Brazil (*n* = 80) [12], Pakistan (*n* = 330) [16] Algeria, Egypt, Jordan, Kuwait, Libya, Palestine, Saudi Arabia and Yemen (*n* = 2936) [11]. They had a cross‐sectional methodological design and the population ranged from 80 [12] to 2936 participants [11]. Previously validated questionnaires were applied, mainly virtually [11, 13, 15, 16] with closed‐ended and open‐ended questions that inquired about sociodemographic variables, knowledge, attitudes, practices and educational needs on domestic violence [11, 12, 13, 15, 16]. Furthermore, the prevalence and experience of detecting abuse, neglect and domestic violence since the dental consultation [13] and the perception of the laws regulating the reporting of these cases [11] (see Table 2). Most of the participants were female (*n* = 2253) and were between 18 and 75 years old.

**Table 2:** Main methodological data extracted from the selected articles.

Author (Publication Year)	Country	Type of study	Sample size	Sample characteristics			Partici‐pants' sector/field of work and special‐ties	Questio‐nnaire used and method of applica‐tion	Evaluated variables	Results		Conclusions
				Sample and sex/gender	Age	Education level				Knowledge, attitudes, and practices	Educa‐tion needs	
Saleem et al. [16]	Pakistan	Descriptive	330 participants	Dentist surgeons, 81.5% women, 18.5% men	23 to 60 years	Undergraduate: 60.6% Specialisation Student: 28.5% Specialist: 10.9%	Public Sector: 33.6% Private Sector: 47.6%	Previously validated and adapted questionnaire.	Sociodemographics characteristics, Knowledge, practices, and experiences regarding domestic violence (DV) cases	55% of dentist surgeons knew that DV cases should be reported, but they were unable to follow the reporting protocols. Women and specialists demonstrated better knowledge of the protocols when suspecting DV	10.6% of participants received training on the care of DV victims	Participants lack adequate knowledge regarding identifying signs, managing, and reporting DV
El Tantawi et al. [11]	Algeria, Egypt, Jordan, Kuwait, Libya, Palestine, Saudi Arabia, and Yemen	Descriptive	2936 participants	Dentists 56.7% women 43.3% men	22 to 40 years	Undergraduate 70.9% and Specialist 29.1%	Public Sector 43.9%, Private 34.2%, and Academic 21.9%	Previously validated and adapted questionnaire	Sociodemographic characteristics Receipt of training Perceived ability to identify DV victims Assessment of the perception of laws for reporting suspected violence Assessment of respondents' attitudes toward reporting DV Intention to report suspected DV to government entities	52.2% of participants had the knowledge to identify clinical signs of DV, which would facilitate reporting. However, the intention to report was associated with the professional's attitude and a negative perception of non‐obligatory reporting	Only 19.3% of participants reported receiving training on the care of DV victims.	The majority of participants intended to report. Nevertheless, it is necessary to train professionals on reporting protocols.
Harris et al. [15]	United States	Descriptive	117 participants	Dentist surgeons 79% women 20% men 1% transgender	25 to 64 years	64% Undergraduate and Associate Degree 35% Bachelor's Degree	Public and Private General Dentistry (69%), Periodontics (2%), Public Health (14%), Paediatric Dentistry (3%), and Orthodontics (3%).	Physician Readiness to Manage Intimate Partner Violence (PREMIS) questionnaire with adapted questions focused on the study's specific sample	Sociodemographic characteristics Education or training on the topic Perceived knowledge Actual knowledge about domestic violence Attitudes and beliefs Practices and policy application	83% of participants reported adequate knowledge about DV. 45% lacked sufficient training to help victims cope with DV. After identifying DV, only 21% of participants provided information to the victim. Only 19% indicated having a protocol for dealing with DV. 61.5% lacked knowledge for identifying DV victims (61.5%) and how to refer them to social services (64%).	58% had attended a lecture or training on DV at some point. 14.5% had witnessed DV training. 8% did not receive training.	Participants reported a high percentage of DV training. However, some were not prepared to approach victims, indicating the need to strengthen knowledge about care protocols
Carvalho et al. [12]	Brazil	Descriptive	80 participants	Dentists 52.5% women 47.5% men	NR	NR	Public network (50%) and private network (50%)	Adapted questionnaire using the instrument for reporting Domestic, Sexual, and/or Other Violence from the Brazilian Ministry of Health. Applied in‐person and completed by the participant.	Sociodemographic characteristics Professional's experience in identifying DV Professional's behaviour in the care of DV victims Education on the topic	Less than 40% of participants identified DV cases during appointments. More than 50% of professionals do not feel prepared to identify signs of DV.	More than 70% did not receive guidance or training on the subject.	The professionals analysed recognise the importance of the dentist in relation to domestic violence but have difficulties in identification and procedures when facing violence.
Drigeard et al. [13]	France	Descriptive	418 participants	General dentist surgeons 57.9% men 42.1% women	NR	100% undergraduate	NR	Self‐developed questionnaire applied virtually via email	Sociodemographic characteristics Assessment of knowledge about DV Estimation of prevalence of abuse and neglect detection Reporting behavior in DV cases Need for education.	59.8% stated they lacked the knowledge and skills to approach DV victims. Only 28% would adopt an attitude in accordance with French legislation in DV cases. 50.3% of participants were not prepared to identify DV cases.	93.9% reported never having attended training on DV.	To help dentist surgeons in the care of DV victims, it is necessary to increase their knowledge and training. Therefore, the inclusion of this topic in their education is recommended.

### 3.3 Risk of Bias Assessment

The risk of bias was low [11, 12, 13, 15, 16]. However, some articles presented deficiencies regarding the calculation and representativeness of the sample [13, 15], but these items did not affect the final evaluation (see Table 3).

**Table 3:** Assessment of bias risk of the selected studies using the Newcastle‐Ottawa Scale adapted for cross‐sectional studies.

	Selection		Comparison		Outcome determination			Score (0−8)
Author (Year)	Case definition	Sample size calculation	Representativeness	Adjustment for confounding factors	Outcome determination	Validity	Non‐response rate	
Saleem et al. [16]	Yes	Yes	Not	Not adjusted	Yes	Yes	Yes	5 Low
El Tantawi et al. [11]	Yes	Yes	Yes	Not adjusted	Yes	Yes	Yes	6 Low
Drigeard et al. [13]	Yes	Not	Not	Not adjusted	Yes	Yes	Yes	4 Low
Carvalho et al. [12]	Yes	Yes	Yes	Not adjusted	Yes	Not	Not	4 Low
Harris et al. [15]	Yes	Not	Not	Not adjusted	Yes	Yes	Yes	4 Low

### 3.4 Summary of Results

#### 3.4.1 Knowledge, Attitudes, Practices Level and Educational Needs

All included studies showed a low level of knowledge, attitudes and practices about domestic violence [11, 12, 13, 15, 16]. In all included articles, the education need on this topic was assessed, and it was reported that there was a high need among dentists to undergo training in protocols for detecting and approaching victims of domestic violence [12, 13, 15]. The studies mainly evaluated domestic violence against women, abuse or neglect [11, 13, 15, 16] and only one study identified situations related to violence against other groups, such as children and the elderly [12]. In one study, a comparison was made between dentists belonging to the public and private health care network, and it was observed that dentists from the public network reported more cases of domestic violence against women [12] Finally, the most identified injuries were bruises on the lips, scratches, burns or scars on the upper and lower extremities, fractured or avulsed teeth and psychological disorders [12].

## 4 Discussion

Although no time restriction was applied in this review, all included studies were published between 2012 and 2021. Notably, across this period, the findings consistently indicate low levels of knowledge, attitudes, and practices among dentists regarding domestic violence. This apparent stability in results over nearly a decade suggests a persistent gap in professional training and highlights a lack of significant progress in preparing dentists to identify and manage cases of domestic violence.

Due to increased prevalence of cases of different types of violence in vulnerable populations, such as women and children, since 2015, the United Nations (UN) has proposed Sustainable Development Goals (SDGs) or Global Goals, which seek to integrate different areas, generating a positive impact on social, environmental and economic sustainability in the world [17]. In this sense, SDGs 3 (Health and Well‐being) and 5 (Gender Equality) aim to reduce maternal, newborn and under‐5 mortality rates, prevent, treat and care for mental health and well‐being, reduce the consumption of alcohol and psychoactive substances [18], in addition to eliminating discrimination and forms of violence against women and children, together with the exclusion of harmful practices, such as early/child marriage and female mutilation [19]. Given this scenario of global mobilisation to confront this public health problem, whose manifestations can occur on a large scale in the head and neck region, there is an urgent need for dentistry to undergo a paradigm shift towards an integral health approach. The first necessary reflection is the one that underpins the present study: how are dentists dealing with the problem, and what knowledge do they have to do so?

The articles selected in this review were published between 2012 [13] and 2021 [16], which indicates that the topic of domestic violence is relevant and current, and that it continues to be a public health problem considering its high rates. The diversity of countries where the studies were conducted: United States [15], France [12, 13], Pakistan [16], Algeria, Egypt, Jordan, Kuwait, Libya, Palestine, Saudi Arabia and Yemen [11] demonstrates that concern about the subject is not focused on a single area or location in the world.

The study by Levin et al. [20]. collected longitudinal evidence for 10 years, indicating that one of the most affected areas in victims of domestic violence was the maxilla, followed by the zygomatic bone and the mandible [20]. Regarding the types of injuries, it is important to highlight that most of them were bruises on the lips, fractured or avulsed teeth, burns or scars [12]. These findings reveal the need for training dentists in identifying injuries caused by domestic violence, with the aim of identifying and reporting cases in a shorter time [20].

There was a wide variation in the prevalence of domestic violence among the included studies conducted in different areas of the world. In the USA, it ranged from 3.8% to 49.6% in 2023 [21]; in Arab countries, where intimate partner violence ranged from 6% to 59% and emotional or psychological violence between 5% and 91% [22], points to underreporting of cases in different countries. In Brazil, in 2019, the domestic violence rate was 7.6%; However, it is known that the country reports a high percentage of femicides, which are preceded by cases of domestic violence [23]. Once again, an alert is raised: where are the cases of DV that are not part of the 7.6% reported?

Regarding the intention to report cases of domestic violence, the study by El Tantawi et al. [11], carried out in 8 Arab countries, showed that most of dentists interviewed had a positive attitude towards reporting and intended to do so when a case was identified during the consultation. However, victim protection services generated a mistaken perception among professionals, who ethically disassociated themselves from the obligation to report and support victims, transferring these responsibilities to these services. Furthermore, DV must be analysed within a sociocultural framework. In various Arab countries, for instance, such DV is not yet legally classified as a crime, and reporting instances remains a significant cultural ‘taboo’ [11]. Consequently, due to their traditional social structures, many of these countries continue to uphold patriarchal family systems. This environment inherently restricts women's individual freedoms as well as their social, economic, and political empowerment.

For this reason, the WHO and the UN have created strategies such as ‘RESPECT Women: Preventing violence against women’, which aims to design, plan, implement, monitor and evaluate programs to prevent violence against women through the integration of different sectors, such as governments, universities, political agents and other stakeholders in the system.

Considering the important role of dentists in identifying and reporting cases of domestic violence, it is suggested that Universities promote links with the secretariats and other local epidemiological surveillance bodies, in order to provide technical training to dentists in relation to both the identification of signs and symptoms of DV and the mandatory reporting of cases in the appropriate information systems.

Among studies included, most used previously validated questionnaires, administered virtually, which was certainly important to improve the reliability of the questions asked to the participants and the results obtained [11, 13, 15, 16]. Since domestic violence is a ‘taboo’ topic, conducting virtual questionnaires facilitates its approach, as well as the anonymity and confidentiality of the indicated responses. However, responses related to attitudes and practices regarding domestic violence can be influenced by what the professional knows as socially correct, generating a response bias. Regarding the methodological quality of the studies selected in this systematic review, they presented a low risk of bias [11, 12, 13, 15, 16]. However, for future studies, it is suggested to calculate the sample and consider the representativeness of the population to be studied, as well as the understanding of oral health care as a collective practice, not restricted to the dental surgeon, but produced within the scope of teamwork, which includes the participation of the dental office staff in the recognition and management of situations of violence.

Although underreported, the data presented in this study are alarming and have a negative impact on female development in each country. In some of them, domestic violence is not even considered a crime and patriarchal systems are evident, with violations of human, marital, sexual and reproductive rights, coercion of freedom of expression, labour inequalities, among others [22]. In all studies included in this systematic review, a low level of knowledge, attitudes and practices regarding domestic violence among dentists was reported [11, 12, 13, 15, 16]. Furthermore, in three included studies, a significant need for education to identify clinical signs and approach victims of domestic violence was reported [12, 13, 15]. This may be related to the fact that dental training, anchored in the biomedical paradigm, is techno‐centred, with an emphasis on procedures, surgical and restorative techniques, and aesthetic and functional apparatus; This can often lead to gaps in the knowledge and practices needed to deal with problems that, although very prevalent, have a social origin, reveal deep emotional dramas, and require different skills from those who deal with them.

The ability to listen, to provide support, and, above all, to have the emotional preparation needed to deal with cases of violence requires minimally reflective and critical training, capable of understanding the origins of this profound problem to the point of being able to talk to the victims, listen to them, and understand them. This points to the need for a curricular arrangement that also allows for an integrative approach to the individual, as well as a multidisciplinary approach that introduces dentistry students to the social reality, epidemiology, and physical manifestations of violence.

A number of interventions have been developed for health professionals with the aim of changing the level of knowledge, approach, and reporting of cases of domestic violence. In Finland, interventions have been implemented using the focus group methodology for health professionals. Interventions have been carried out in dentists using different strategies, such as booklets, refresher lectures, practical groups for case studies and identification of injuries, and assessment of health care received by victims and perpetrators of DV. However, the generation of good practices in victim care and case reporting requires a reflective analysis by the professionals who make up the multidisciplinary team supporting victims. In order to carry out interventions that modify the educational needs on this topic, it is recommended to develop continuing education processes that promote organisational impact and that are carried out in the long term. In the literature, there are reports on the negative effect of limited time for victim care, few resources and the lack of commitment of the professional team on the final results of interventions on DV case care.

This study goes even deeper into the proposal of comprehensiveness and proposes above all that the construction of the curriculum takes into account specifically the prevalence of violence in society, especially DV, its manifestation in the head and neck region as well as the role of dentists in this approach, and offers future professionals specific content related to the identification of injuries, notification of cases in the respective information systems and referral of victims to the health care network. Furthermore, in undergraduate programs, disciplines focused on in‐depth knowledge of the problem from a psychosocial perspective are essential for the proper understanding and support of victims.

De Jesus Santos Nascimento et al. [24] published a systematic review with a similar focus and included studies up to 2018. However, the strengths of this systematic review were the execution of a rigorous search and selection protocol, where different keywords and Boolean connectors in English, Spanish and Portuguese were included for the primary search and the development of secondary search strategies in the list of references of selected studies and grey literature. The application of eligibility criteria and assessment of methodological quality by two standardised researchers and the intervention of a third researcher in cases of disagreement are also important. The inclusion of studies conducted in different countries allows us to demonstrate the cultural diversity and sociodemographic differences among the professionals studied, which would allow the generalisation of the results.

In order to conduct studies that complement this research line, further studies should investigate variables such as the intention to report various forms of violence and the reluctance to do so stemming from apprehension regarding patient loss or professional exposure. Additionally, it is essential to examine dentists' comfort levels when discussing these issues, and the role of office staff's involvement in the process. Furthermore, dental schools analyze the different approaches and perspectives on DV within their curricula, promoting for substantive reforms that enhance awareness among future dentists regarding this issue. Finally, qualitative studies involving interviews or focus groups with dentists would also be relevant to investigate feelings during care for victims, and barriers to reporting cases would also be relevant.

## 5 Conclusion

Scientific evidence suggests that dentists have a low level of knowledge, attitudes and practices regarding the identification of clinical signs and reporting of cases of domestic violence. In addition, they showed a high need for education on this topic.

## Funding

The authors have nothing to report.

## Ethics Statement

This study did not require approval from a research ethics committee, as it was a systematic review based exclusively on data already published in the scientific literature and did not involve human participants or identifiable personal data.

## Conflicts of Interest

The authors declare no conflicts of interest.

## Data Availability

All data underlying this article are included within the article and its Supporting Information Materials.
